# The Role of Lipophagy in the Development and Treatment of Non-Alcoholic Fatty Liver Disease

**DOI:** 10.3389/fendo.2020.601627

**Published:** 2021-02-01

**Authors:** Aldo Grefhorst, Ivo P. van de Peppel, Lars E. Larsen, Johan W. Jonker, Adriaan G. Holleboom

**Affiliations:** ^1^ Department of Experimental Vascular Medicine, Amsterdam University Medical Centers, location AMC, Amsterdam, Netherlands; ^2^ Section of Molecular Metabolism and Nutrition, Department of Pediatrics, University of Groningen, University Medical Center Groningen, Groningen, Netherlands; ^3^ Department of Vascular Medicine, Amsterdam University Medical Centers, location AMC, Amsterdam, Netherlands

**Keywords:** non-alcoholic fatty liver disease, lipophagy, lipid droplet, autophagy, lipid homeostasis

## Abstract

Non-alcoholic fatty liver disease (NAFLD) or metabolic (dysfunction) associated liver disease (MAFLD), is, with a global prevalence of 25%, the most common liver disorder worldwide. NAFLD comprises a spectrum of liver disorders ranging from simple steatosis to steatohepatitis, fibrosis, cirrhosis and eventually end-stage liver disease. The cause of NAFLD is multifactorial with genetic susceptibility and an unhealthy lifestyle playing a crucial role in its development. Disrupted hepatic lipid homeostasis resulting in hepatic triglyceride accumulation is an hallmark of NAFLD. This disruption is commonly described based on four pathways concerning 1) increased fatty acid influx, 2) increased *de novo* lipogenesis, 3) reduced triglyceride secretion, and 4) reduced fatty acid oxidation. More recently, lipophagy has also emerged as pathway affecting NAFLD development and progression. Lipophagy is a form of autophagy (i.e. controlled autolysosomal degradation and recycling of cellular components), that controls the breakdown of lipid droplets in the liver. Here we address the role of hepatic lipid homeostasis in NAFLD and specifically review the current literature on lipophagy, describing its underlying mechanism, its role in pathophysiology and its potential as a therapeutic target.

## Introduction

Non-alcoholic fatty liver disease (NAFLD) comprises a spectrum of liver diseases that is characterized by an increased hepatic triglyceride (TG) content (i.e. steatosis) in the absence of excessive alcohol use. The NAFLD disease spectrum ranges from simple steatosis *via* non-alcoholic steatohepatitis (NASH) with or without fibrosis, to advanced irreversible scarring (cirrhosis), hepatocellular carcinoma and ultimately end stage liver disease (1, 2). Recently, it has been suggested to change the terminology of NAFLD/NASH to metabolic (dysfunction) associated liver disease (MAFLD) to more adequately reflect current knowledge and pathophysiology of the disease (3, 4). For clarity, we will use the term NAFLD in this review.

Hepatic steatosis is generally benign and is considered the primary step in the pathogenesis of NAFLD. Progressive stages of NAFLD, e.g. steatohepatitis and hepatic fibrosis, occur when excessive lipid accumulation overwhelms the capacity of the liver to store, secrete and oxidize fatty acids (5). In this lipotoxic environment, necroinflammation and fibrogenesis can occur. Various lipid species may drive this lipotoxicity, especially lysophospholipids and diacylglycerol (6). Therefore, controlling general hepatic lipid accumulation is essential to prevent or reverse progression of NAFLD.

Driven by an unhealthy diet and sedentary lifestyle, NAFLD is considered to be the hepatic manifestation of the metabolic syndrome (MetS), a condition that is defined by the presence of a combination of cardiovascular risk factors including abdominal obesity, insulin resistance, hypertension and atherogenic dyslipidemia (7–10). Parallel to the rise in obesity, the prevalence of NAFLD is increasing worldwide and a recent study suggests that about 25% of the worldwide population has some stage of NAFLD (11, 12). The proportion of NAFLD patients that develops steatohepatitis is around 40%, contributing to liver-specific and overall mortality among NAFLD patients (12). This renders NAFLD not only as a clinical, but also an economic burden. NAFLD is estimated to account for more than 35 billion euro of medical costs annually in the United Kingdom, France, Germany and Italy combined and more than 100 billion dollars per year in the United States (12). While there are great efforts towards development of drugs to treat NAFLD (13), clinical applicability is still rather limited and current NAFLD therapy is mainly centered around lifestyle changes (8, 14).

**Figure 1:**
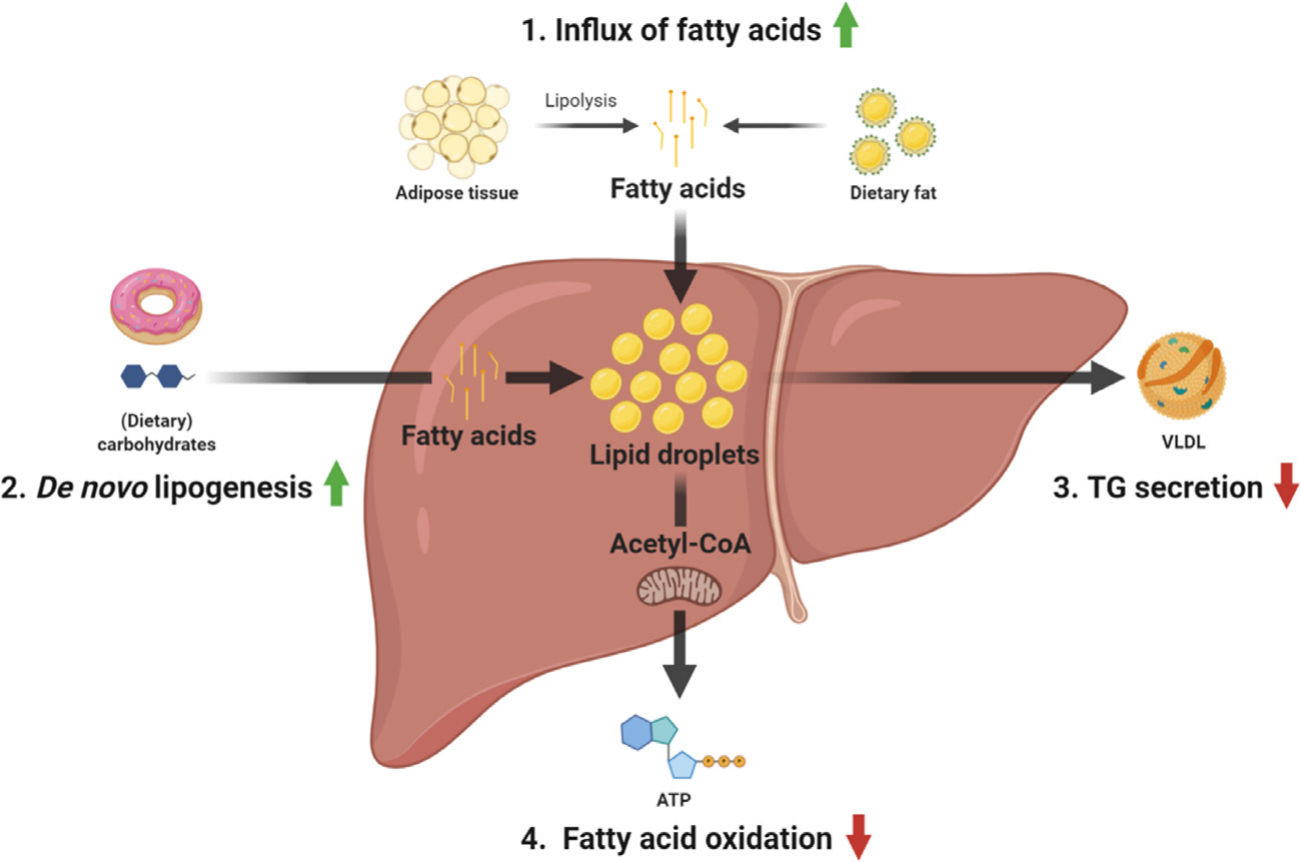
Overview of the main pathways contributing to TG accumulation in NAFLD. 1) fatty acid uptake, 2) *de novo* lipogenesis, 3) TG secretion, and 4) fatty acid oxidation. ATP, adenosine triphosphate; TG, triglyceride; VLDL, very-low-density lipoprotein.

Four main pathways relating to hepatic lipid metabolism have been identified to contribute to hepatic TG accumulation (**Figure 1**), namely 1) uptake of circulating fatty acids derived from the diet or adipose tissue; 2) hepatic fatty acid synthesis also known as *de novo* lipogenesis (DNL); 3) secretion of TGs in the form of very low density lipoprotein (VLDL) particles; and 4) fatty acid oxidation (FAO) (15). Recently, turnover of lipid droplets (LDs) by lipophagy, a form of autophagy specifically involved in the degradation of LDs has been identified as a novel pathway that also contributes to NAFLD.

Several reviews have discussed the role of lipophagy in NAFLD (16–18). However, these studies did not provide a complete overview of lipophagy in the context of hepatic lipid homeostasis and lacked recent insight and assessment of the potential of lipophagy as a therapeutic target to prevent or treat NAFLD. The aim of this review is, therefore, to provide an expert opinion on the role of lipophagy in NAFLD development and progression as well as its potential in treatment. We searched the PubMed database for “(NAFLD OR liver) AND (lipophagy OR autophagy)” and selected studies published in the English language between 2005 and mid 2020 in peer-reviewed journals. Here, we will discuss the studies we deemed relevant and of sufficient quality. To establish the role of lipophagy in NAFLD in an adequate context, we describe the four established pathways contributing to hepatic TG accumulation in section *The Four Established Pathways That Result in Excessive Hepatic TG Accumulation* and provide general information on autophagy and lipophagy in sections *Autophagy* and *Lipophagy: the Fifth Pathway Involved in NAFLD*, respectively. The last two sections discuss the pre-clinical (section *Pre-Clinical Studies on the Role of Lipophagy in NAFLD*) and clinical (section *Clinical Studies*) studies that specifically addressed the role of lipophagy in NAFLD.

## The Four Established Pathways that Result in Excessive Hepatic Triglyceride Accumulation

Although a healthy liver normally does not store large amounts of neutral lipids, it plays a central and crucial role in whole body lipid homeostasis, as it is the main site for fatty acid synthesis, metabolism and transport. Disturbances in any one of these processes can contribute to NAFLD development and progression.

### Hepatic Incorporation of FFA Into Triglyceride: A Link With Adipose Tissue

The adipose tissue (AT) depots are the main sites of TG storage in the mammalian body. Upon increased energy demand, e.g. during fasting or exercise, TGs stored in the AT will be liberated as fatty acids through lipolysis and transported in the circulation as free fatty acids (FFA) bound to albumin. Dietary lipids are released by the intestinal tract into the circulation as components of chylomicrons. Upon TG hydrolysis by lipoprotein lipase (LPL), fatty acids from the chylomicrons can also be taken up by the liver. In general, the liver takes up around 25% of all FFAs that pass it, and this is primarily mediated by the fatty acid transporter Cluster of differentiation 36 (CD36) (19, 20). Insulin plays an important role in regulating FFA levels by inhibiting AT lipolysis. In this way insulin favors fat storage after a meal, whereas energy is liberated from fat stores during fasting. During insulin resistance (IR), insulin is no longer able to suppress lipolysis and this results in a chronically high flux of FFAs to the liver and this is considered to be one of the main drivers of NAFLD (21–24). Stable isotope studies have shown that AT-derived fatty acids are indeed the dominant source of hepatic TG accumulation in NAFLD (25, 26). In addition, lipodystrophic disorders in which a patient is incapable of storing fatty acids in the AT depots are also associated with ectopic lipid accumulation, i.e. (severely) elevated TG concentrations in the liver and to a lesser extent in the muscles (27), underlining the important connection between AT and liver TG accumulation.

Various rodent as well as human studies demonstrated that inflammation of AT, i.e. accumulation of activated macrophages, exacerbates or is associated with more severe NAFLD and steatohepatitis (28–32). The AT derived hormone adiponectin has been proposed to be the crucial link in this association. Adiponectin can enhance hepatic fatty acid oxidation (33, 34) and suppresses hepatic DNL (35). Macrophages in the AT secrete proinflammatory factors such as tumor necrosis factor-α (TNF-α) and interleukin-6 (IL-6) that suppress the production of adiponectin by adipocytes (36, 37). Therefore, lower adiponectin levels due to increased AT inflammation can contribute to hepatic TG accumulation, NAFLD development, and progression.

### Increased De Novo Lipogenesis

Aside from TG synthesis from externally derived fatty acids, the liver also synthesizes fatty acids *de novo* which are subsequently esterified into TGs. While DNL is not very abundant in a healthy liver, it contributes up to 26% of hepatic TG concentrations in patients with NAFLD (26). Dietary carbohydrates are the most important source of TGs derived by DNL and this process is stimulated by insulin. Under insulin sensitive conditions, insulin directs carbohydrates to the peripheral tissues (e.g. muscle and AT) and stimulates storage of hepatic glucose as glycogen as well as DNL. Under insulin resistant conditions, insulin is not able to direct carbohydrates away from the liver resulting in increased hepatic TG storage, glycogenesis and DNL. Altogether, the excess hepatic carbohydrate concentrations in concert with the elevated DNL will result in more TGs in the liver. As such, hepatic insulin resistance contributes to the elevated hepatic TG concentrations, partly by higher DNL (38).

### Disturbances in Hepatic Triglyceride Secretion

Hepatic TGs can be secreted by the liver as part of VLDL particles. These lipoprotein particles, which consist of lipidated Apolipoprotein B (ApoB) carry TGs to peripheral tissues where they are hydrolyzed by LPL and the released fatty acids provide fuel for the generation of energy. Although reduced VLDL secretion can contribute to excessive hepatic TG accumulation, this occurs only in rare, genetically affected patients. Examples are hypobetalipoproteinemia caused by mutations in the gene encoding *ApoB* and in abetalipoproteinemia due to mutations in the gene encoding microsomal TG transport protein (*MTP*) that is responsible for the lipidation of ApoB (39, 40). More commonly, NAFLD is associated with increased VLDL secretion due to elevated TG availability for the intrahepatic ApoB lipidation process (23), resulting in the characteristic mixed hyperlipidemia in which both VLDL and low-density lipoprotein (LDL) are increased that is often observed in insulin resistant and diabetic patients with NAFLD. Indeed, since insulin has been shown to suppress VLDL secretion (41, 42), insulin resistance coinciding with NAFLD also enhances VLDL secretion.

### Disturbances in Hepatic Fatty Acid Oxidation

Hepatic fatty acid oxidation (FAO) encompasses fatty acid breakdown into shorter chains by a series of dehydrogenases in mitochondria and, for very long chain fatty acids, the peroxisomes (43). For this, the fatty acids first pass the mitochondrial membrane by the aid of carnitine palmitoyltransferase-1 (CPT1) and CPT2, enzymes localized in the outer and inner mitochondrial membrane, respectively. While CPT1 adds carnitine to the fatty acyl-CoA molecule to facilitate mitochondrial transport, CPT2 removes carnitine (44). FAO disorders have been shown to result in steatosis, especially in the fasted state (45). Pharmacological blockade of CPT1 for example resulted in severe hepatic steatosis in fasted mice (46), while mice that lack peroxisome proliferator-activated receptor α (PPARα), a nuclear receptor controlling expression of almost all FAO genes, also develop hepatic steatosis along with high plasma FFA concentrations, hypoglycemia and hypoketonemia when fasted (47, 48). In humans, FAO defects are also associated with hepatic steatosis and hepatomegaly (49).

## Autophagy

Autophagy is the dedicated process by which cells dispose of and recycle unwanted, abnormal or malfunctional structures such as cytosolic organelles and macromolecules. It clears these structures by directing them to the lysosomes for degradation. The term autophagy (self-eating) was already coined in the 19th century to describe survival in periods of starvation (50). However, it was the Belgian biochemist De Duve who was the first to use autophagy in its present cellular context (51), i.e. the dynamic recycling system essential for cellular renovation and homeostasis (52).

Autophagy can be classified into three subcategories; macroautophagy, microautophagy, and chaperone-mediated autophagy (CMA) (53). Macroautophagy is the most active and common form in mammalian cells and is therefore almost synonymously used with autophagy. Crucial in autophagy is the formation of autophagosomes from membranes that isolate cell structure(s) that need to be degraded. These structures (or cargo) are recognized by the autophagosome membrane-bound microtubule-associated protein 1 light chain 3 (LC3-1, the mammalian homologue of the yeast protein ATG8). Subsequently, autophagy related cargo adaptors p62, NDP52, Optineurin, and NBR1 are involved in the adherence of the cargo to the autophagosome membrane (54). Upon the capture of the cargo, late endosomes and lysosomes fuse with the autophagosome to form the autolysosome in order to complete the degradation process for further metabolism through the ER (52, 53). In the process of autolysosome formation, members of the lysosome associated membrane protein (LAMP) family are crucial, especially LAMP2 (55). The Vacuolar H+-ATPase (V-ATPase) is a large multi-subunit protein complex that is required for acidification, an essential step in the autophagic process because an acidic environment activates enzymes essential to degrade biological materials (56).

A large number of proteins encoded by autophagy-related genes (ATGs) play crucial roles in controlling the autophagy process. For instance, ATG9 is a transmembrane protein that cycles between the trans-Golgi network and endosomes that carry membranes required for expansion of the autophagosomal membrane (57). Among other proteins that play important roles in the elongation of the autophagosomal membrane are ATG5, ATG7, ATG10, ATG12 and ATG16L1 (58, 59).

As mentioned, LC3 is crucial in the autophagic process. The LC3 precursor is cleaved by ATG4 which results in the cytosolic isoform LC3-I (60). In a reaction that involves ATG7 and ATG3, LC3-I can be conjugated to phosphatidylethanolamine (PE) to form LC3-II (61, 62), a protein that targets to the elongated autophagosome membrane. This association of LC3-II with the autophagosome makes it an excellent protein marker to investigate autophagy (63).

Autophagosome formation is negatively regulated by the (mammalian) target of rapamycin (mTOR) (64) which activity is inhibited under starvation conditions (65).

## Lipophagy: The Fifth Pathway Involved in Non-Alcoholic Fatty Liver Disease

A specific form of autophagy is lipophagy which mediates the liberation of lipids stored in LDs. Lipophagy is a relatively recent discovery compared to the four pathways described above. However, disturbances in lipophagy have been linked to NAFLD and hepatic TG accumulation (66–68) and this process can therefore be considered the fifth pathway controlling NAFLD development.

### Lipid Droplets

In order to protect against lipotoxicity, several organs including the liver store neutral lipids such as TGs in specialized single membrane vacuoles known as LDs (69). LDs are predominantly generated at the ER and increase in size by lipid transfer from the ER (70) or by fusion with other LDs (71). LDs consist of a single phospholipid membrane filled with neutral lipids and harbor a variety of lipolytic enzymes and other LD regulatory proteins. Adipose triglyceride lipase (ATGL), hormone sensitive lipase (HSL), di- and monoglyceride lipase (DGL and MGL, respectively) facilitate the hydrolysis of TGs into fatty acids (71, 72). Additionally, members of the perilipin family (PLIN1-PLIN5) are colocalized on the LD-membrane where they serve a regulatory function for lipolysis. PLIN2 and PLIN5 are highly expressed in the liver and contribute to the formation and regulation of lipolytic activity at the LDs membrane (73). The regulatory role of PLIN2 was demonstrated when *Plin2* knockout mice did not develop hepatic steatosis in response to a high-fat diet (74). Their regulatory role was associated to an occupational function on the LDs, reducing the special availability for lipolytic enzymes (75). Hepatic PLIN5 protein synthesis and activity were both induced in mice by fasting, and signals *via* sirtuin-1 (SIRT1) to promote autophagy and reduce hepatic inflammatory injury under starvation (76). In addition, PLIN5 was shown to regulated the phosphorolytic state of lipolytic enzymes (ATGL, HSL, and MGL), and thereby altering their lipolytic activity (75). These examples of dysfunction of LD-associated proteins elegantly illustrate the regulatory and inducible axis between LDs, cytosolic lipolysis, and lipophagy.

Another class of proteins found on the LD membrane are the members of the cell death-inducing DFFA-like effector (CIDE) protein family of which CIDEB is constitutively expressed in the liver where it is thought to control VLDL secretion (77). Livers of *Cideb* deficient mice contained less TGs and had smaller LDs than their wild type littermates when challenged with a high-fat diet (78).

### Lipophagy and Liquid Droplet Turnover

LDs and their content are among the structures that are degraded in the lipophagic process. As such, lipophagy is important to liberate fatty acids from the TGs stored in the LDs. A recent study reported that lipophagy-derived fatty acids undergo a cycle of efflux followed by reuptake before a subsequent reincorporation into cellular LDs (79). However, if lipophagy is associated with reduced hepatic lipid content, it is likely that some of the liberated fatty acids will be directed towards FAO. In general it is considered that lysosomal acid lipase (LAL) hydrolyzes the TGs to generate fatty acids (80, 81). The presence of a second lipolytic enzyme, e.g. ATGL, necessarily contributes to the degradation of the LD (82, 83).

### A Role of Lipophagy in Non-Alcoholic Fatty Liver Disease?

Several studies have investigated the role of genes/proteins involved lipophagy and its association with NAFLD development and progression (summarized in **Table 1**). In this section, we provide a general overview of the evidence on the role of lipophagy in NAFLD and in the following paragraphs we discuss the pre-clinical and clinical studies, respectively, in more detail.

**Table 1:** Lipophagy (associated) genes/proteins that are associated with NAFLD.

Gene/protein	Description	NAFLD association^1^	Type of evidence	Ref.
IRGM	Autophagy-related member of the interferon-inducible GTPase family	Positive	Human genetic association and *in vitro* studies	(84, 85)
Rubicon	Negative regulator of autophagosome-lysosome fusion	Positive	Human liver biopsies and mouse studies	(86)
CD36	Fatty acid transporter	Positive	Mouse and *in vitro* studies	(87)
FIP200	Subunit of autophagy related complex 1	Positive	Mouse study	(88)
LC3A/B-II ATG16L1	Autophagy proteins	Positive	Human liver biopsies	(89)
p62	Autophagy protein	Positive	Human liver biopsies, mouse and *in vitro* studies	(66, 67, 90)
LAMP3	Involved in autolysosome fusion process	Positive	Human liver biopsies, mouse and *in vitro* studies	(91, 92)
ATP6A1 ATP6A2 TMEM199 CCDC115	Factors in the V-ATPase complex	Negative	Human genetic association, mouse and *Drosophila* studies	(93–95)
VMA21	Involved in V-ATPase assembly	Negative	Human genetic association study	(96)
GNMT	Catalyzes synthesis of N-methylglycine using SAMe	Negative	Human (serum protein) association and mouse studies	(97)
ATG5 ATG7 ATG14	Autophagy proteins	Negative	Mouse and *in vitro* studies	(98–101)
PLD1	Catalyzes synthesis of phosphatic acid species that play a role in mTOR signaling	Negative	Mouse study	(102)
SOD1	Protects against oxidative stress	Negative	Mouse study	(103)
TFEB	Regulator of autophagy	Negative	Human liver biopsies and mouse studies	(104–106)
LC3 II/I	Autophagy protein	Negative	Rat and *in vitro* studies	(107, 108)

^1^Positive association: higher concentrations, expression, and/or activity are associated with increased NAFLD development or occurrence. Negative association: higher concentrations, expression, and/or activity are associated with reduced NAFLD development or occurrence.

Mechanistic studies assessing lipophagy to modulate disease progression in NAFLD are still limited. Several human association studies have demonstrated that mutations in autophagy-related genes increase the risk of NAFLD development. A recent study using both pre-clinical models and patient material demonstrated that advanced stage NAFLD is associated with greater impairments of hepatic autophagy (66) and an association was also found between the autophagy-related GTPase family M (*IRGM*) gene and increased susceptibility of NAFLD in obese children (84). *In vitro* experiments in HepG2 cells revealed that *IRGM* knockdown inhibited the autophagic flux and increased LD content while overexpression of *IRGM* decreased LD content, highlighting its role in lipophagy.

Another example of dysfunctioning lipophagic proteins leading to NAFLD are assembly factors of the V-ATPase complex, which is involved in acidification of organelles. Although mutations in four V-ATPase genes, *ATP6AP1*, *ATP6AP2*, *TMEM199*, and *CCDC115*, present with different clinical characteristics, steatosis—ranging from mild to severe steatohepatitis including liver failure - is an important hallmark of this group of inborn errors of metabolism (93–95). A recent study on *VMA21*, a gene important to V-ATPase assembly, showed that mutations leading to V-ATPase dysfunction presented as an autophagic myopathy (96). Additional *in vitro* and *in vivo* experiments confirmed that these mutations lead to LD accumulation in autolysosomes and subsequently steatohepatitis and elevated transaminases.

It has been suggested that in some patients with severe NAFLD, decreased expression of the enzyme glycine N-methyltransferase (GNMT) results in an increased serum concentration of methionine and its metabolite S-adenosylmethionine (SAMe), both known inactivators of autophagy which might lead to impaired lipophagy (97). Alternatively, elevated levels of Rubicon, a negative regulator of autophagosome-lysosome fusion, were found in liver samples taken from patients with NAFLD (86). Taken together, these results demonstrate the role of autophagic genes and thus lipophagy in (hereditary predisposition to) NAFLD.

## Pre-Clinical Studies on the Role of Lipophagy in Non-Alcoholic Fatty Liver Disease

Several murine models have been used to investigate the mechanistic role of specific autophagic proteins in NAFLD development. The first study that described mechanistic involvement of lipophagy in NAFLD showed that pharmacological inhibition of autophagy with 3-methyladenine or knockdown of either one of the autophagy genes *ATG5* or *ATG7* increased hepatic TG concentrations (98). While Liu et al. (99) already demonstrated that hepatic autophagy is suppressed in conditions associated with NAFLD, i.e. insulin resistance and hyperinsulinemia, Yang et al. (100) addressed the role of liver specific autophagy in obesity, insulin resistance and NAFLD more comprehensively using several models. In the latter study, downregulation of hepatic *Atg7* was found in high-fat diet induced obese mice as well as in genetic models of obesity (*ob/ob* and *db/db* mice). Hyperinsulinemia was not causal as treatment of *ob/ob* mice with streptozotocin reduced insulin concentrations by 5-fold but did not restore hepatic *Atg7* expression. In *ob/ob* mice, restoration of *Atg7* expression improved the metabolic phenotype which could be completely prevented by blocking the downstream autophagy mediator Atg5. Knockdown of *Atg14* produced similar results on hepatic TG accumulation in mice (101).

Several genes have been shown to affect both hepatic lipid content and lipophagy. When mice were fed a high-fat diet they developed NAFLD with a reduced hepatic lipophagic rate as well as an elevated hepatic expression of the fatty acid transporter *CD36* (87). This was supported by the observation that knocking down *Cd36* increased lipophagy *in vitro* and *in vivo* while overexpression of *CD36* in HepG2 and Huh7 cells reduced lipophagy. Phospholipase D1 (PLD1) is an enzyme that hydrolyzes phosphatidylcholine to produce phosphatic acid species that play a role in mTOR signaling (109). *Pld1* knockout mice display an impairment in autophagic flux and have increased NAFLD development when placed on a high-fat diet (102). Knockdown of superoxide dismutase (SOD) 1, an enzyme involved in protection against oxidative stress, in mice resulted in increased hepatic TG accumulation despite low visceral adiposity (103). This effect was attributed to reduced lipophagy because hepatic levels of p62 were increased. While p62 is essential for autophagy and lipophagy as it connects cargo with autophagosomes, elevated levels usually represent accumulation/aggregation due decreased autophagy (110).

In line with reduced lipophagy in NAFLD, a series of pre-clinical studies demonstrated that induction of lipophagy can attenuate NAFLD. Various interventions have been reported to stimulate autophagy/lipophagy and thereby attenuate or reduce development of NAFLD in mice (summarized in **Table 2**). In high-fat diet-induced NAFLD in mice, 4 week caffeine treatment not only enhanced the autophagic pathway but also significantly reduced hepatosteatosis (111). Similar effects have been reported with several herbal extracts [e.g. resveratrol, dioscin, capsaicin, (-)-Epigallocatechin-3-gallate] that have been suggested as novel therapeutic approaches to manage NAFLD (112–114). Also quercetin, a flavonoid found in various fruits and vegetables, anthocyanins from sweet cherries, and trehalose, a naturally occurring disaccharide derived from cocoons, have been reported to activate hepatic autophagy and reduce hepatic lipid content (115, 116, 124, 125). In mice, long-term exercise protects against high-fat diet-induced hepatic lipid accumulation at least partly by increasing the autophagic flux (117). Fasting, intermittent fasting, caloric restriction and caloric restriction mimetic drugs all induce autophagy in various tissues including the liver and could therefore also be used as a strategy to reduce hepatic steatosis (118–120).

**Table 2:** Pharmacological and other interventions that stimulate autophagy/lipophagy and reduced NAFLD development in mice.

Compound/intervention	Ref.
Caffeine	(111)
Resveratrol	(112)
Dioscin	(113)
Capsaicin	(112)
(-)-Epigallocatechin-3-gallate	(114)
Quercetin	(115)
Trehalose	(116)
Exercise	(117)
Fasting	(118)
Intermittent fasting	(119)
Caloric restriction	(118)
Caloric restriction mimetics	(120)
Rapamycin	(121)
Carbamazepine	(121)
TFEB agonists	(104, 105)
Celecoxib	(107, 108)
Thyroid hormone	(122)
JNK inhibitor	(123)

Direct pharmacological induction of hepatic autophagy using rapamycin and carbamazepine was shown to be protective in high-fat diet-induced NAFLD in mice (121). Direct evidence of the role of autophagy in controlling NAFLD comes from the studies of Wang et al. (104) who developed three potent agonists for Transcription Factor EB (TFEB), a master regulator of lysosomal biogenesis and autophagy. When mice on a high-fat diet were treated with these agonists, both hepatic steatosis and features of the MetS decreased. Another compound, referred to as MSL, that activates TFEB, also lowered hepatic TG concentrations of *ob/ob* and diet-induced obese mice (105). The importance of TFEB was also demonstrated by the observation that ezetimibe, a Niemann-Pick C1-Like 1 (NPC1L1) inhibitor reducing cholesterol absorption, improved steatohepatitis *in vitro* and *in vivo via* AMPK activation and TFEB nuclear translocation (106). Celecoxib, a nonsteroidal anti-inflammatory drug (NSAID), reduced development of diet-induced hepatic steatosis and inflammation in rodent models, potentially *via* restoring autophagic flux through increased LC3 II/I (107, 108). Thyroid hormone, an important regulator of metabolism, increases hepatic lipid catabolism at least partially through stimulation of autophagy (122). Reduced thyroid hormone secretion or sensitivity could therefore also contribute to NAFLD pathophysiology.

Despite the large number of studies describing a link between lipophagy and NAFLD, some debate still exists on the exact role of (members of) the autophagic and lipophagic pathway in NAFLD (126, 127). One study showed that in high-fat diet fed rats, treatment with a c-Jun N-terminal kinase (JNK) inhibitor (SP600125) decreased expression of autophagy-associated genes and reduced insulin resistance and NAFLD development (123). Ma et al. (88) argued that not autophagy impairment in the liver *per se* induces or attenuates NAFLD development but that it sensitizes the liver to other damaging triggers. They found that mice with a liver-specific deficiency of focal adhesion kinase family kinase-interacting protein 200 kDa (FIP200, also known as Rb1cc1), a core subunit of the mammalian autophagy related 1 complex, were actually protected from high-fat diet-induced liver TG accumulation. However, stimulation of liver autophagy by exposure to lipopolysaccharides, sensitized the FIP200 hepatic deficient mice to liver injury.

## Clinical Studies

Although the link between NAFLD and lipophagy/autophagy has been explored extensively in pre-clinical studies, not much human data are available. Lin et al. (84) genotyped 832 obese children (aged 6–18 years) of East Asian descent and found that a variant in the lipophagy associated gene *IRGM* increased the chance to develop NAFLD, diagnosed using an ultrasonography based scoring pattern described by Chan et al. (85), by approximately 2-fold. When comparing biopsies from NAFL (n=2), steatohepatitis (n=3) and healthy (n=4) livers, Lee et al. (89) found that protein levels of LC3A/B-II and ATG16L1 were increased in NAFLD livers with steatohepatitis while other autophagic factors such as LC3A/B-I and p62 were not different. None of the measured autophagy proteins were different between NAFLD and healthy livers. However, other studies did find significant differences in other autophagic proteins in liver biopsies of NAFLD patients, especially increased p62 levels. Fukuo et al. (90) found accumulation of p62 in 65% of liver biopsies of 22 NAFLD patients. Fukushima et al. (67) investigated liver biopsies of 31 NAFLD patients with NAFLD activity scores (NAS) ranging from 2 to 8 and fibrosis scores from 0 to 4 and compared these to five healthy control liver biopsies. Aggregation of p62 was found in about 88% of the NAFLD biopsies while it was undetectable in healthy controls. The number of hepatocytes with p62 aggregation correlated positively with the number of autophagic vesicles and various NAFLD severity outcomes (including NAS, fibrosis, and serum alanine aminotransferase levels). An even more recent study confirmed this finding by immunohistochemistry staining of liver biopsies of 59 patients with steatohepatitis, In these biopsies, there was increased accumulation of p62 clusters and alterations in autophagy-related gene expression that correlated with NAS and fibrosis stage (66).

Nuclear expression of the master regulator of autophagy TFEB was lower in human liver samples with both simple steatosis (n=11) and steatohepatitis (n=9) compared to healthy controls (n=12) (106). Combined with the previously described pre-clinical data (128), TFEB could be a promising therapeutic target for NAFLD. While not prescribed as a TFEB modulator, ezetimibe has been studied as a potential treatment for NAFLD. There are some human studies that show improvements in some NAFLD (surrogate) outcomes upon ezetimibe treatment (129). However, more and larger trials are needed to substantiate these effects. Additionally, whether the potential beneficial effects of ezetimibe are the result of its effect on lipophagy or (intestinal) cholesterol absorption also remains elusive.

The LAMP family plays a critical role in the autolysosome fusion process (55). Recently it was found that LAMP3 protein expression was higher in the livers of patients with NAFLD (n=4) compared to healthy controls (n=3) (91). Further *in vitro* experiments revealed that LAMP3 overexpression resulted in higher expression of lipogenic genes. More evidence for a link between LAMP and NAFLD was demonstrated in livers of 24 NAFLD patients with varying degrees of steatosis (mild, moderate, severe) where a reduction of LAMP2A expression and other positive regulators of autophagy such as PLIN5 was found (92).

Altogether, we conclude that although clinical studies on lipophagy and NAFLD are limited and observational in nature, current evidence suggests a similar link as has been reported in pre-clinical studies. A reduction in various genes and proteins involved lipophagy correlates with the presence of steatosis and potentially NAFLD severity.

## Conclusions and Future Perspectives

**Figure 2:**
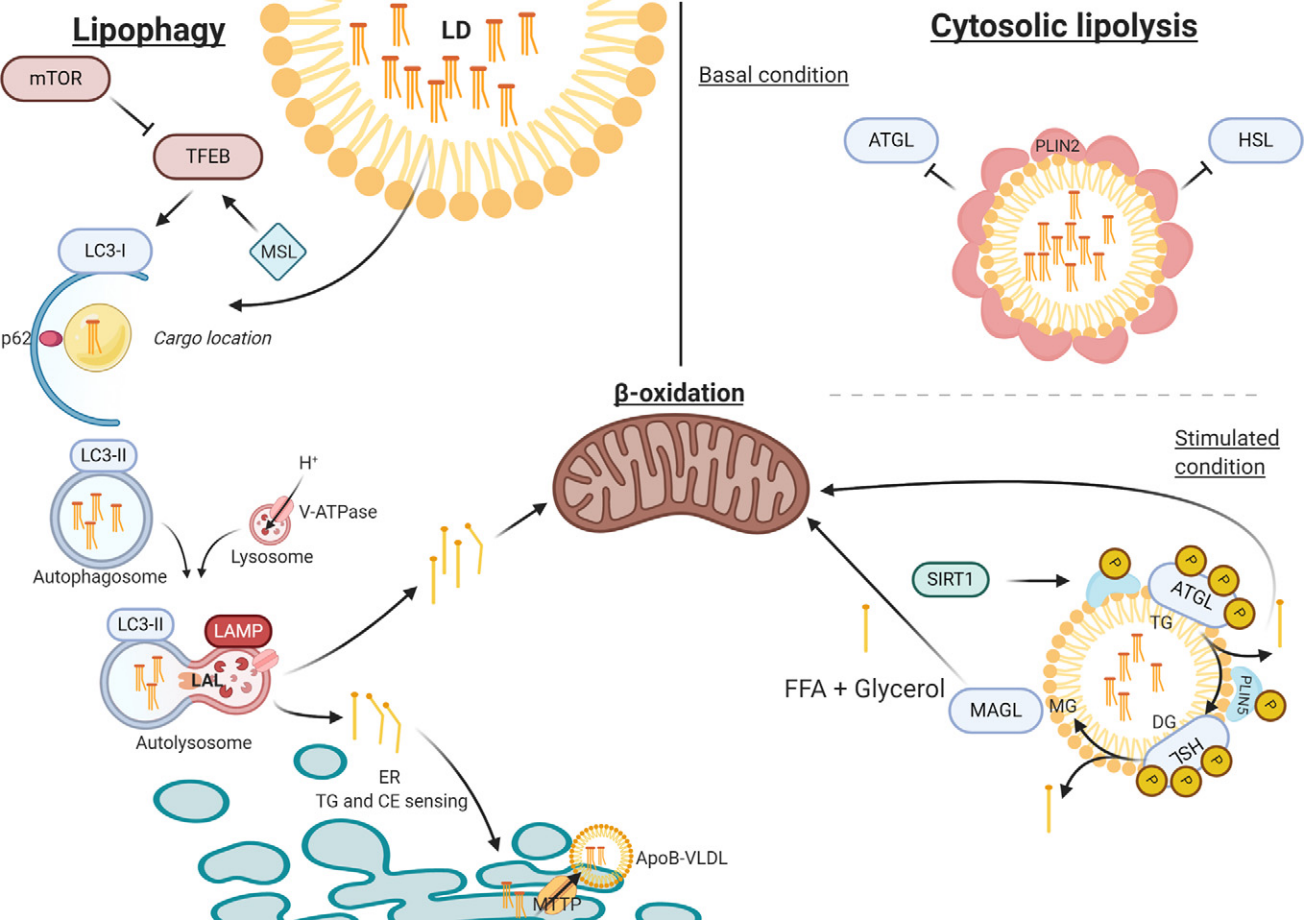
The process of lipophagy. *Lipophagy* – Key regulator mTOR inhibits TFEB in autophagy induction. MSL induces TFEB that upregulates autophagy. LC3-I assembles at the autophagosome envelope elongating the bi-layer. P62 secures ‘chunks’ of the LD as lipid-cargo in the lumen of the autophagosome. After LC3-I is spliced by ATG4 to LC3-II, the autophagosome closes carrying its cargo. Upon fusion with the acidic lysosome (LAMP and V-ATPase driven), LAL can hydrolyze TG and CE into FFA and free cholesterol, respectively. FFA are directed towards either beta-oxidation or to the ER for further metabolism. *Cytosolic Lipolysis* – At basal conditions, LDs are covered in PLIN2 molecules that regulate the accessibility for lipolytic enzymes. When stimulated PLIN2 releases and grants access at the membrane for ATGL and HSL (bound to PLIN5), which activate under phosphorylation. When stimulated, SIRT1 activates CMA to release PLIN2 and replace PLIN5 at the LD membrane, thereby mediating the lipolytic activity through autophagy. ATP, adenosine triphosphate; ATG4, autophagy-regulating protease 4; ATGL, adipose triglyceride lipase; CE, cholesteryl esters; CMA, chaperone mediated autophagy; ER, endoplasmic reticulum; FFA, free fatty acid; HSL, hormone sensitive lipase; LAL, lysosome acid lipase; LAMP, lysosomal-associated membrane protein; LD, lipid droplet; mTOR, mammalian target of rapamycin; PLIN, perilipin; TFEB, transcription factor EB; TG, triglyceride; V-ATPase, Vacuolar-type-H^+^-ATPase.

In this review we have described the four classical pathways that contribute to hepatic TG accumulation in NAFLD (**Figure 1**) and specifically addressed a recently discovered novel pathway that concerns the lysosomal turnover of LDs, known as lipophagy (**Figure 2**). We have summarized data from numerous *in vitro*, *in vivo* and observational human studies that demonstrate that lipophagy is an important pathway contributing to disrupted hepatic lipid homeostasis in NAFLD. Lipophagy is a complex process and impediments at various stages can contribute to NAFLD development. In turn, NAFLD itself might lead to impairments in lipophagy, resulting in a vicious cycle promoting disease progression.

Despite its potential to reduce TG accumulation and lipotoxicity, relatively little of the enormous drug development effort for NAFLD is focused on lipophagy (13). There are two main explanations for this. Firstly, lipophagy is a recent discovery and while the number of studies assessing it have dramatically increased over the last few years, the exact role of lipophagy in the pathophysiology of NAFLD and as a therapeutic target are still incompletely understood. While recent insights from genetic disorders such as IRGM deficiency and the V-ATPase assembly factor defects show promising results on the role of lipophagy in (certain) NAFLD patients, human evidence for the relevance of lipophagy in NAFLD is scarce and observational. Secondly, specific and targetable regulators and chaperones need to be identified in order to reduce chances of undesired autophagic side-effects.

When these caveats are addressed, selective de-repression or stimulation of lipophagy may turn out to be an effective and important treatment option in the growing global epidemic of NAFLD and its complications.

## Author Contributions

All authors contributed to the article and approved the submitted version.

## Conflict of Interest

The authors declare that the research was conducted in the absence of any commercial or financial relationships that could be construed as a potential conflict of interest.

## References

[1] Brunt EM Pathology of nonalcoholic fatty liver disease. Nat Rev Gastroenterol Hepatol (2010) 7(4):195–203. 10.1038/nrgastro.2010.21 20195271

[2] Brunt EM Wong VW-S Nobili V Day CP Sookoian S Maher JJ Nonalcoholic fatty liver disease. Nat Rev Dis Prim (2015) 1:15080. 10.1038/nrdp.2015.80 27188459

[3] Eslam M Newsome PN Sarin SK Anstee QM Targher G Romero-Gomez M A new definition for metabolic dysfunction-associated fatty liver disease: an international expert consensus statement. J Hepatol (2020) 73(1):202–9. 10.1016/j.jhep.2020.07.045 32278004

[4] Eslam M Sanyal AJ George J MAFLD: a consensus-driven proposed nomenclature for metabolic associated fatty liver disease. Gastroenterology (2020) 158(7):1999–2014. 10.1053/j.gastro.2019.11.312 32044314

[5] Tacke F Luedde T Trautwein C Inflammatory Pathways in Liver Homeostasis and Liver Injury. Clin Rev Allergy Immunol (2009) 36(1):4–12. 10.1007/s12016-008-8091-0 18600481

[6] Koliaki C Szendroedi J Kaul K Jelenik T Nowotny P Jankowiak F Adaptation of hepatic mitochondrial function in humans with non-alcoholic fatty liver is lost in steatohepatitis. Cell Metab (2015) 21(5):739–46. 10.1016/j.cmet.2015.04.004 25955209

[7] Huang PL A comprehensive definition for metabolic syndrome. Dis Models Mechanisms (2009) 2(5–6):231–7. 10.1242/dmm.001180 PMC2675814 19407331

[8] European Association for the Study of the Liver (EASL) European Association for the Study of Diabetes (EASD), European Association for the Study of Obesity (EASO). EASL-EASD-EASO Clinical Practice Guidelines for the management of non-alcoholic fatty liver disease. J Hepatol (2016) 64(6):1388–402. 10.1016/j.jhep.2015.11.004 27062661

[9] Hardy T Oakley F Anstee QM Day CP Nonalcoholic Fatty Liver Disease: Pathogenesis and Disease Spectrum. Annu Rev Pathol (2016) 11:451–96. 10.1146/annurev-pathol-012615-044224 26980160

[10] Diehl AM Day C Cause, Pathogenesis, and Treatment of Nonalcoholic Steatohepatitis. New Engl J Med (2017) 377(21):2063–72. 10.1056/NEJMra1503519 29166236

[11] Satapathy SK Sanyal AJ Epidemiology and Natural History of Nonalcoholic Fatty Liver Disease. Semin Liver Dis (2015) 35(3):221–35. 10.1055/s-0035-1562943 26378640

[12] Younossi ZM Koenig AB Abdelatif D Fazel Y Henry L Wymer M Global epidemiology of nonalcoholic fatty liver disease-Meta-analytic assessment of prevalence, incidence, and outcomes. Hepatol (Baltimore Md) (2016) 64(1):73–84. 10.1002/hep.28431 26707365

[13] Francque S Vonghia L Pharmacological Treatment for Non-alcoholic Fatty Liver Disease. Adv Ther (2019) 36(5):1052–74. 10.1007/s12325-019-00898-6 PMC6824365 30888594

[14] Chalasani N Younossi Z Lavine JE Charlton M Cusi K Rinella M The diagnosis and management of nonalcoholic fatty liver disease: practice guidance from the American Association for the Study of Liver Diseases. Hepatology (2018) 67(1):328–57. 10.1002/hep.29367 28714183

[15] Willebrords J Pereira IVA Maes M Crespo Yanguas S Colle I van den Bossche B Strategies, models and biomarkers in experimental non-alcoholic fatty liver disease research. Prog Lipid Res (2015) 59:106–25. 10.1016/j.plipres.2015.05.002 PMC4596006 26073454

[16] Ueno T Komatsu M Autophagy in the liver: functions in health and disease. Nat Rev Gastroenterol Hepatol (2017) 14(3):170–84. 10.1038/nrgastro.2016.185 28053338

[17] Schulze RJ McNiven MA Lipid droplet formation and lipophagy in fatty liver disease. Semin Liver Dis (2019) 39(3):283–90. 10.1055/s-0039-1685524 PMC8718013 31041790

[18] Kounakis K Chaniotakis M Markaki M Tavernarakis N Emerging roles of lipophagy in health and disease. Front Cell Dev Biol (2019) 7:185. 10.3389/fcell.2019.00185 31552248 PMC6746960

[19] Iozzo P Turpeinen AK Takala T Oikonen V Bergman J Grönroos T Defective liver disposal of free fatty acids in patients with impaired glucose tolerance. J Clin Endocrinol Metab (2004) 89(7):3496–502. 10.1210/jc.2003-031142 15240637

[20] He J Lee JH Febbraio M Xie W The emerging roles of fatty acid translocase/CD36 and the aryl hydrocarbon receptor in fatty liver disease. Exp Biol Med (Maywood NJ) (2011) 236(10):1116–21. 10.1258/ebm.2011.011128 21885479

[21] Sanyal AJ Campbell-Sargent C Mirshahi F Rizzo WB Contos MJ Sterling RK Nonalcoholic steatohepatitis: association of insulin resistance and mitochondrial abnormalities. Gastroenterology (2001) 120(5):1183–92. 10.1053/gast.2001.23256 11266382

[22] Korenblat KM Fabbrini E Mohammed BS Klein S Liver, muscle, and adipose tissue insulin action is directly related to intrahepatic triglyceride content in obese subjects. Gastroenterology (2008) 134(5):1369–75. 10.1053/j.gastro.2008.01.075 PMC2629391 18355813

[23] Fabbrini E Mohammed BS Magkos F Korenblat KM Patterson BW Klein S Alterations in adipose tissue and hepatic lipid kinetics in obese men and women with nonalcoholic fatty liver disease. Gastroenterology (2008) 134(2):242–31. 10.1053/j.gastro.2007.11.038 PMC2705923 18242210

[24] Lomonaco R Ortiz-Lopez C Orsak B Webb A Hardies J Darland C Effect of adipose tissue insulin resistance on metabolic parameters and liver histology in obese patients with nonalcoholic fatty liver disease. Hepatol (Baltimore Md) (2012) 55(5):1389–97. 10.1002/hep.25539 22183689

[25] Barrows BR Parks EJ Contributions of different fatty acid sources to very low-density lipoprotein-triacylglycerol in the fasted and fed states. J Clin Endocrinol Metab (2006) 91(4):1446–52. 10.1210/jc.2005-1709 16449340

[26] Donnelly KL Smith CI Schwarzenberg SJ Jessurun J Boldt MD Parks EJ Sources of fatty acids stored in liver and secreted via lipoproteins in patients with nonalcoholic fatty liver disease. J Clin Investigat (2005) 115(5):1343–51. 10.1172/JCI23621 PMC1087172 15864352

[27] Polyzos SA Perakakis N Mantzoros CS Fatty liver in lipodystrophy: A review with a focus on therapeutic perspectives of adiponectin and/or leptin replacement. Metabol: Clin Experiment (2019) 96:66–82. 10.1016/j.metabol.2019.05.001 31071311

[28] du Plessis J van Pelt J Korf H Mathieu C van der Schueren B Lannoo M Association of Adipose Tissue Inflammation With Histologic Severity of Nonalcoholic Fatty Liver Disease. Gastroenterology (2015) 149(3):635–48. 10.1053/j.gastro.2015.05.044 26028579

[29] Duval C Thissen U Keshtkar S Accart B Stienstra R van Boekschoten M Adipose tissue dysfunction signals progression of hepatic steatosis towards nonalcoholic steatohepatitis in C57BL/6 mice. Diabetes (2010) 59(12):3181–91. 10.2337/db10-0224 PMC2992781 20858684

[30] Patsouris D Li PP Thapar D Chapman J Olefsky JM Neels JG Ablation of CD11c-positive cells normalizes insulin sensitivity in obese insulin resistant animals. Cell Metab (2008) 8(4):301–9. 10.1016/j.cmet.2008.08.015 PMC2630775 18840360

[31] Weisberg SP Hunter D Huber R Lemieux J Slaymaker S Vaddi K CCR2 modulates inflammatory and metabolic effects of high-fat feeding. J Clin Investigat (2006) 116(1):115–24. 10.1172/JCI24335 PMC1307559 16341265

[32] Cimini FA Barchetta I Ciccarelli G Leonetti F Silecchia G Chiapetta C Adipose tissue remodelling in obese subjects is a determinant of presence and severity of fatty liver disease. Diabetes Metab Res Rev (2020) 37(1):e3358. 10.1002/dmrr.3358 32469428

[33] Bugianesi E Pagotto U Manini R Vanni E Gastaldelli A de Iasio R Plasma adiponectin in nonalcoholic fatty liver is related to hepatic insulin resistance and hepatic fat content, not to liver disease severity. J Clin Endocrinol Metab (2005) 90(6):3498–504. 10.1210/jc.2004-2240 15797948

[34] Tomas E Tsao TS Saha AK Murrey HE Cc ZCc SI I Enhanced muscle fat oxidation and glucose transport by ACRP30 globular domain: acetyl-CoA carboxylase inhibition and AMP-activated protein kinase activation. Proc Natl Acad Sci United States America (2002) 99(25):16309–13. 10.1073/pnas.222657499 PMC138607 12456889

[35] Xu A Wang Y Keshaw H Xu LY Lam KS Cooper GJ The fat-derived hormone adiponectin alleviates alcoholic and nonalcoholic fatty liver diseases in mice. J Clin Investigat (2003) 112(1):91–100. 10.1172/JCI200317797 PMC162288 12840063

[36] Weisberg SP McCann D Desai M Rosenbaum M Leibel RL Ferrante AW Jr. Obesity is associated with macrophage accumulation in adipose tissue. J Clin Investigat (2003) 112(12):1796–808. 10.1172/JCI200319246 PMC296995 14679176

[37] Zoico E Garbin U Olioso D Mazzali G Fratta Pasini AM Di Francesco V The effects of adiponectin on interleukin-6 and MCP-1 secretion in lipopolysaccharide-treated 3T3-L1 adipocytes: role of the NF-kappaB pathway. Int J Mol Med (2009) 24(6):847–51. 10.3892/ijmm_00000302 19885628

[38] Smith GI Shankaran M Yoshino M Schweitzer GG Chondronikola M Beals JW Insulin resistance drives hepatic de novo lipogenesis in nonalcoholic fatty liver disease. J Clin Investigat (2020) 130(3):1453–60. 10.1172/JCI134165 PMC7269561 31805015

[39] Tanoli T Yue P Yablonskiy D Schonfeld G Fatty liver in familial hypobetalipoproteinemia: roles of the APOB defects, intra-abdominal adipose tissue, and insulin sensitivity. J Lipid Res (2004) 45(5):941–7. 10.1194/jlr.M300508-JLR200 14967820

[40] Berriot-Varoqueaux N Aggerbeck LP Samson-Bouma M Wetterau JR The role of the microsomal triglygeride transfer protein in abetalipoproteinemia. Annu Rev Nutrition (2000) 20:663–97. 10.1146/annurev.nutr.20.1.663 10940349

[41] Phung TL Roncone A Jensen KL Sparks CE Sparks JD Phosphoinositide 3-kinase activity is necessary for insulin-dependent inhibition of apolipoprotein B secretion by rat hepatocytes and localizes to the endoplasmic reticulum. J Biol Chem (1997) 272(49):30693–702. 10.1074/jbc.272.49.30693 9388205

[42] Durrington PN Newton RS Weinstein DB Steinberg D Effects of insulin and glucose on very low density lipoprotein triglyceride secretion by cultured rat hepatocytes. J Clin Investigat (1982) 70(1):63–73. 10.1172/JCI110604 PMC370227 7045162

[43] Cherkaoui-Malki M Surapureddi S El-Hajj HI Vamecq J Andreoletti P Hepatic steatosis and peroxisomal fatty acid beta-oxidation. Curr Drug Metab (2012) 13(10):1412–21. 10.2174/138920012803762765 22978396

[44] Ramsay RR Gandour RD van der Leij FR Molecular enzymology of carnitine transfer and transport. Biochim Biophys Acta (2001) 1546(1):21–43. 10.1016/S0167-4838(01)00147-9 11257506

[45] Rinaldo P Matern D Bennett MJ Fatty acid oxidation disorders. Annu Rev Physiol (2002) 64:477–502. 10.1146/annurev.physiol.64.082201.154705 11826276

[46] Grefhorst A Hoekstra J Derks TG Ouwens DM Baller JF Havinga R Acute hepatic steatosis in mice by blocking beta-oxidation does not reduce insulin sensitivity of very-low-density lipoprotein production. Am J Physiol Gastrointest Liver Physiol (2005) 289(3):G592–8. 10.1152/ajpgi.00063.2005 15817811

[47] Bandsma RHJ van Dijk TH ter HAtA Kok T Reijngoud D-J Staels B Hepatic de novo synthesis of glucose 6-phosphate is not affected in peroxisome proliferator-activated receptor alpha-deficient mice but is preferentially directed toward hepatic glycogen stores after a short term fast. J Biol Chem (2004) 279(10):8930–7. 10.1074/jbc.M310067200 14688286

[48] Guerre-Millo M Rouault C Poulain P André J Poitout V Peters JM PPAR-alpha-null mice are protected from high-fat diet-induced insulin resistance. Diabetes (2001) 50(12):2809–14. 10.2337/diabetes.50.12.2809 11723064

[49] Saudubray JM Martin D de Lonlay P Touati G Poggi-Travert F Bonnet D Recognition and management of fatty acid oxidation defects: a series of 107 patients. J Inherited Metab Dis (1999) 22(4):488–502. 10.1023/A:1005556207210 10407781

[50] Ktistakis NT In praise of M. Anselmier who first used the term ‘autophagie’ in 1859. Autophagy (2017) 13(12):2015–7. 10.1080/15548627.2017.1367473 PMC5788564 28837378

[51] Sabatini DD Adesnik M Christian de Duve: Explorer of the cell who discovered new organelles by using a centrifuge. Proc Natl Acad Sci United States America (2013) 110(33):13234–5. 10.1073/pnas.1312084110 PMC3746853 23924611

[52] Mizushima N Komatsu M Autophagy: renovation of cells and tissues. Cell (2011) 147(4):728–41. 10.1016/j.cell.2011.10.026 22078875

[53] Meijer AJ Codogno P Regulation and role of autophagy in mammalian cells. Int J Biochem Cell Biol (2004) 36(12):2445–62. 10.1016/j.biocel.2004.02.002 15325584

[54] Rubinsztein DC Shpilka T Elazar Z Mechanisms of autophagosome biogenesis. Curr Biol (2012) 22(1):R29–34. 10.1016/j.cub.2011.11.034 22240478

[55] Wang K Autophagy and apoptosis in liver injury. Cell Cycle (Georgetown Tex) (2015) 14(11):1631–42. 10.1080/15384101.2015.1038685 PMC4614283 25927598

[56] Kissing S Saftig P Haas A Vacuolar ATPase in phago(lyso)some biology. Int J Med Microbiol (2018) 308(1):58–67. 10.1016/j.ijmm.2017.08.007 28867521

[57] Young ARJ Chan EYW Hu XW Köchl R Crawshaw SG High S Starvation and ULK1-dependent cycling of mammalian Atg9 between the TGN and endosomes. J Cell Sci (2006) 119(Pt 18):3888–900. 10.1242/jcs.03172 16940348

[58] Mizushima N Sugita H Yoshimori T Ohsumi Y A new protein conjugation system in human. The counterpart of the yeast Apg12p conjugation system essential for autophagy. J Biol Chem (1998) 273(51):33889–92. 10.1074/jbc.273.51.33889 9852036

[59] Mizushima N Kuma A Kobayashi Y Yamamoto A Matsubae M Takao T Mouse Apg16L, a novel WD-repeat protein, targets to the autophagic isolation membrane with the Apg12-Apg5 conjugate. J Cell Sci (2003) 116(Pt 9):1679–88. 10.1242/jcs.00381 12665549

[60] Tanida I Sou Y Ezaki J Minematsu-Ikeguchi N Ueno T Kominami E HsAtg4B/HsApg4B/autophagin-1 cleaves the carboxyl termini of three human Atg8 homologues and delipidates microtubule-associated protein light chain 3- and GABAA receptor-associated protein-phospholipid conjugates. J Biol Chem (2004) 279(35):36268–76. 10.1074/jbc.M401461200 15187094

[61] Tanida I Ueno T Kominami E LC3 conjugation system in mammalian autophagy. Int J Biochem Cell Biol (2004) 36(12):2503–18. 10.1016/j.biocel.2004.05.009 PMC7129593 15325588

[62] Kabeya Y Mizushima N Ueno T Yamamoto A Kirisako T Noda T LC3, a mammalian homologue of yeast Apg8p, is localized in autophagosome membranes after processing. EMBO J (2000) 19(21):5720–8. 10.1093/emboj/19.21.5720 PMC305793 11060023

[63] Klionsky DJ Abeliovich H Agostinis P Agrawal DK Aliev G Askew DS Guidelines for the use and interpretation of assays for monitoring autophagy in higher eukaryotes. Autophagy (2008) 4(2):151–75. 10.4161/auto.5338 PMC2654259 18188003

[64] Walker SA Ktistakis NT Autophagosome Biogenesis Machinery. J Mol Biol (2020) 432(8):2449–24612. 10.1016/j.jmb.2019.10.027 31705882

[65] Leprivier G Rotblat B How does mTOR sense glucose starvation? AMPK is the usual suspect. Cell Death Discovery (2020) 6:27. 10.1038/s41420-020-0260-9 PMC7176732 32351714

[66] Carotti S Aquilano K Zalfa F Ruggiero S Valentini F Zingariello M Lipophagy Impairment Is Associated With Disease Progression in NAFLD. Front Physiol (2020) 11:850. 10.3389/fphys.2020.00850 32765301 PMC7380071

[67] Fukushima H Yamashina S Arakawa A Taniguchi G Aoyama T Uchiyama A Formation of p62-positive inclusion body is associated with macrophage polarization in non-alcoholic fatty liver disease. Hepatol Res (2018) 48(9):757–67. 10.1111/hepr.13071 29473277

[68] Patrick AD Lake BD Deficiency of an acid lipase in Wolman’s disease. Nature (1969) 222(5198):1067–8. 10.1038/2221067a0 5787090

[69] Gross DA Silver DL Cytosolic lipid droplets: from mechanisms of fat storage to disease. Crit Rev Biochem Mol Biol (2014) 49(4):304–26. 10.3109/10409238.2014.931337 25039762

[70] Gross DA Zhan C Silver DL Direct binding of triglyceride to fat storage-inducing transmembrane proteins 1 and 2 is important for lipid droplet formation. Proc Natl Acad Sci United States America (2011) 108(49):19581–6. 10.1073/pnas.1110817108 PMC3241795 22106267

[71] Walther TC Farese RV Lipid droplets and cellular lipid metabolism. Annu Rev Biochem (2012) 81:687–714. 10.1146/annurev-biochem-061009-102430 22524315 PMC3767414

[72] Young SG Zechner R Biochemistry and pathophysiology of intravascular and intracellular lipolysis. Genes Dev (2013) 27(5):459–84. 10.1101/gad.209296.112 PMC3605461 23475957

[73] Itabe H Yamaguchi T Nimura S Sasabe N Perilipins: a diversity of intracellular lipid droplet proteins. Lipids Health Dis (2017) 16(1):83. 10.1186/s12944-017-0473-y 28454542 PMC5410086

[74] McManaman JL Bales ES Orlicky DJ Jackman M MacLean PS Cain S Perilipin-2-null mice are protected against diet-induced obesity, adipose inflammation, and fatty liver disease. J Lipid Res (2013) 54(5):1346–59. 10.1194/jlr.M035063 PMC3622329 23402988

[75] Sztalryd C Brasaemle DL The perilipin family of lipid droplet proteins: Gatekeepers of intracellular lipolysis. Biochim Biophys Acta Mol Cell Biol Lipids (2017) 1862(10 Pt B):1221–32. 10.1016/j.bbalip.2017.07.009 PMC5595658 28754637

[76] Zhang E Cui W Lopresti M Mashek MT Najt CP Hu H Hepatic PLIN5 signals via SIRT1 to promote autophagy and prevent inflammation during fasting. J Lipid Res (2020) 61(3):338–50. 10.1194/jlr.RA119000336 PMC7053830 31932301

[77] Ye J Li JZ Liu Y Li X Yang T Ma X Cideb, an ER- and lipid droplet-associated protein, mediates VLDL lipidation and maturation by interacting with apolipoprotein B. Cell Metab (2009) 9(2):177–90. 10.1016/j.cmet.2008.12.013 19187774

[78] Li JZ Ye J Xue B Qi J Zhang J Zhou Z Cideb regulates diet-induced obesity, liver steatosis, and insulin sensitivity by controlling lipogenesis and fatty acid oxidation. Diabetes (2007) 56(10):2523–32. 10.2337/db07-0040 17646209

[79] Cui W Sathyanarayan A Lopresti M Aghajan M Chen C Mashek DG Lipophagy-derived fatty acids undergo extracellular efflux via lysosomal exocytosis. Autophagy (2020) 1–16. 10.1080/15548627.2020.1728097 PMC8032247 32070194

[80] Gluchowski NL Becuwe M Walther TC Farese RV Lipid droplets and liver disease: from basic biology to clinical implications. Nat Rev Gastroenterol Hepatol (2017) 14(6):343–55. 10.1038/nrgastro.2017.32 PMC6319657 28428634

[81] Martinez-Lopez N Singh R Autophagy and Lipid Droplets in Liver. Annu Rev Nutrition (2015) 35:215–37. 10.1146/annurev-nutr-071813-105336 PMC7909712 26076903

[82] Schulze RJ Drižytė K Casey CA McNiven MA Hepatic Lipophagy: New Insights into Autophagic Catabolism of Lipid Droplets in the Liver. Hepatol Commun (2017) 1(5):359–69. 10.1002/hep4.1056 PMC5669271 29109982

[83] Zechner R Madeo F Kratky D Cytosolic lipolysis and lipophagy: two sides of the same coin. Nat Rev Mol Cell Biol (2017) 18(11):671–84. 10.1038/nrm.2017.76 28852221

[84] Lin Y-C Chang P-F Lin H-F Liu K Chang M-H Ni Y-H Variants in the autophagy-related gene IRGM confer susceptibility to non-alcoholic fatty liver disease by modulating lipophagy. J Hepatol (2016) 65(6):1209–16. 10.1016/j.jhep.2016.06.029 27417217

[85] Chan DFY Li AM Chu WCW Chan MHM Wong EMC Liu EKH Hepatic steatosis in obese Chinese children. Int J Obes Related Metab Disord (2004) 28(10):1257–63. 10.1038/sj.ijo.0802734 15278103

[86] Tanaka S Hikita H Tatsumi T Sakamori R Nozaki Y Sakane S Rubicon inhibits autophagy and accelerates hepatocyte apoptosis and lipid accumulation in nonalcoholic fatty liver disease in mice. Hepatol (Baltimore Md) (2016) 64(6):1994–2014. 10.1002/hep.28820 27637015

[87] Li Y Yang P Zhao L Chen Y Zhang X Zeng S CD36 plays a negative role in the regulation of lipophagy in hepatocytes through an AMPK-dependent pathway. J Lipid Res (2019) 60(4):844–55. 10.1194/jlr.M090969 PMC6446711 30662007

[88] Ma D Molusky MM Song J Hu C-R Fang F Rui C Autophagy deficiency by hepatic FIP200 deletion uncouples steatosis from liver injury in NAFLD. Mol Endocrinol (Baltimore Md) (2013) 27(10):1643–54. 10.1210/me.2013-1153 PMC4061382 23960084

[89] Lee S Kim S Hwang S Cherrington NJ Ryu D-Y Dysregulated expression of proteins associated with ER stress, autophagy and apoptosis in tissues from nonalcoholic fatty liver disease. Oncotarget (2017) 8(38):63370–81. 10.18632/oncotarget.18812 PMC5609929 28968997

[90] Fukuo Y Yamashina S Sonoue H Arakawa A Nakadera E Aoyama T Abnormality of autophagic function and cathepsin expression in the liver from patients with non-alcoholic fatty liver disease. Hepatol Res (2014) 44(9):1026–36. 10.1111/hepr.12282 24299564

[91] Liao X Song L Zhang L Wang H Tong Q Xu J LAMP3 regulates hepatic lipid metabolism through activating PI3K/Akt pathway. Mol Cell Endocrinol (2018) 470:160–7. 10.1016/j.mce.2017.10.010 29056532

[92] Ma SY Sun KS Zhang M Zhou X Zheng XH Tian SY Disruption of Plin5 degradation by CMA causes lipid homeostasis imbalance in NAFLD. Liver Int (2020) 40(10):2427–38. 10.1111/liv.14492 32339374

[93] Jansen JC Cirak S van Scherpenzeel M Timal S Reunert J Rust S CCDC115 Deficiency Causes a Disorder of Golgi Homeostasis with Abnormal Protein Glycosylation. Am J Hum Genet (2016) 98(2):310–21. 10.1016/j.ajhg.2015.12.010 PMC4746332 26833332

[94] Rujano MA Cannata Serio M Panasyuk G Péanne R Reunert J Rymen D Mutations in the X-linked ATP6AP2 cause a glycosylation disorder with autophagic defects. J Exp Med (2017) 214(12):3707–29. 10.1084/jem.20170453 PMC5716037 29127204

[95] Jansen EJR Timal S Ryan M Ashikov A van Scherpenzeel M Graham LA ATP6AP1 deficiency causes an immunodeficiency with hepatopathy, cognitive impairment and abnormal protein glycosylation. Nat Commun (2016) 7:11600. 10.1038/ncomms11600 27231034 PMC4894975

[96] Cannata Serio M Graham LA Ashikov A Larsen LE Raymond K Timal S sMutations in the V-ATPase assembly factor VMA21 cause a congenital disorder of glycosylation with autophagic liver disease. Hepatol (Baltimore Md) (2020). 10.1002/hep.31218 PMC7483274 32145091

[97] Zubiete-Franco I García-Rodríguez JL Martínez-Uña M Martínez-Lopez N Woodhoo A Juan VG-D Methionine and S-adenosylmethionine levels are critical regulators of PP2A activity modulating lipophagy during steatosis. J Hepatol (2016) 64(2):409–18. 10.1016/j.jhep.2015.08.037 PMC4718902 26394163

[98] Singh R Kaushik S Wang Y Xiang Y Novak I Komatsu M Autophagy regulates lipid metabolism. Nature (2009) 458(7242):1131–5. 10.1038/nature07976 PMC2676208 19339967

[99] Liu H-Y Han J Cao SY Hong T Zhuo D Shi J Hepatic autophagy is suppressed in the presence of insulin resistance and hyperinsulinemia: inhibition of FoxO1-dependent expression of key autophagy genes by insulin. J Biol Chem (2009) 284(45):31484–92. 10.1074/jbc.M109.033936 PMC2781544 19758991

[100] Yang L Li P Fu S Calay ES Hotamisligil GS Defective hepatic autophagy in obesity promotes ER stress and causes insulin resistance. Cell Metab (2010) 11(6):467–78. 10.1016/j.cmet.2010.04.005 PMC2881480 20519119

[101] Xiong X Tao R DePinho RA Dong XC The autophagy-related gene 14 (Atg14) is regulated by forkhead box O transcription factors and circadian rhythms and plays a critical role in hepatic autophagy and lipid metabolism. J Biol Chem (2012) 287(46):39107–14. 10.1074/jbc.M112.412569 PMC3493951 22992773

[102] Hur JH Park S-Y Dall’Armi C Lee JS di Paolo G Lee H-Y Phospholipase D1 deficiency in mice causes nonalcoholic fatty liver disease via an autophagy defect. Sci Rep (2016) 6:39170. 10.1038/srep39170 27976696 PMC5156943

[103] Kurahashi T Hamashima S Shirato T Lee J Homma T Kang ES An SOD1 deficiency enhances lipid droplet accumulation in the fasted mouse liver by aborting lipophagy. Biochem Biophys Res Commun (2015) 467(4):866–71. 10.1016/j.bbrc.2015.10.052 26474701

[104] Wang C Niederstrasser H Douglas PM Lin R Jaramillo J Li Y Small-molecule TFEB pathway agonists that ameliorate metabolic syndrome in mice and extend C. elegans lifespan. Nat Commun (2017) 8(1):2270. 10.1038/s41467-017-02332-3 29273768 PMC5741634

[105] Lim H Lim Y-M Kim KH Jeon YE Park K Kim J A novel autophagy enhancer as a therapeutic agent against metabolic syndrome and diabetes. Nat Commun (2018) 9(1):1438. 10.1038/s41467-018-03939-w 29650965 PMC5897400

[106] Kim SH Kim G Han DH Lee M Kim I Kim B Ezetimibe ameliorates steatohepatitis via AMP activated protein kinase-TFEB-mediated activation of autophagy and NLRP3 inflammasome inhibition. Autophagy (2017) 13(10):1767–81. 10.1080/15548627.2017.1356977 PMC5640190 28933629

[107] Liu C Liu L Zhu H-D Sheng J-Q Wu X-L He X-X Celecoxib alleviates nonalcoholic fatty liver disease by restoring autophagic flux. Sci Rep (2018) 8(1):4108. 10.1038/s41598-018-22339-0 29515134 PMC5841322

[108] Chen J Liu D Bai Q Song J Guan J Gao J Celecoxib attenuates liver steatosis and inflammation in non-alcoholic steatohepatitis induced by high-fat diet in rats. Mol Med Rep (2011) 4(5):811–6. 10.3892/mmr.2011.501 21643627

[109] Yoon MS Du G Backer JM Frohman MA Chen J Class III PI-3-kinase activates phospholipase D in an amino acid-sensing mTORC1 pathway. J Cell Biol (2011) 195(3):435–47. 10.1083/jcb.201107033 PMC3206351 22024166

[110] Rusten TE Stenmark H p62, an autophagy hero or culprit? Nat Cell Biol (2010) 12(3):207–9. 10.1038/ncb0310-207 20190829

[111] Sinha RA Farah BL Singh BK Siddique MM Li Y Wu Y Caffeine stimulates hepatic lipid metabolism by the autophagy-lysosomal pathway in mice. Hepatol (Baltimore Md) (2014) 59(4):1366–80. 10.1002/hep.26667 23929677

[112] Liu C Liao J-Z Li P-Y Traditional Chinese herbal extracts inducing autophagy as a novel approach in therapy of nonalcoholic fatty liver disease. World J Gastroenterol (2017) 23(11):1964–73. 10.3748/wjg.v23.i11.1964 PMC5360637 28373762

[113] Liu M Xu L Yin L Qi Y Xu Y Han X Potent effects of dioscin against obesity in mice. Sci Rep (2015) 5:7973. 10.1038/srep12183 25609476 PMC4302319

[114] Chen C Liu Q Liu L Hu Y-Y Feng Q Potential Biological Effects of (-)-Epigallocatechin-3-gallate on the Treatment of Nonalcoholic Fatty Liver Disease. Mol Nutr Food Res (2018) 62(1):1700483. 10.1002/mnfr.201700483 PMC6120134 28799714

[115] Zhu X Xiong T Liu P Guo X Xiao L Zhou F Quercetin ameliorates HFD-induced NAFLD by promoting hepatic VLDL assembly and lipophagy via the IRE1a/XBP1s pathway. Food Chem Toxicol (2018) 114:52–60. 10.1016/j.fct.2018.02.019 29438776

[116] DeBosch BJ Heitmeier MR Mayer AL Higgins CB Crowley JR Kraft TE Trehalose inhibits solute carrier 2A (SLC2A) proteins to induce autophagy and prevent hepatic steatosis. Sci Signaling (2016) 9(416):ra21. 10.1126/scisignal.aac5472 PMC4816640 26905426

[117] Pi H Liu M Xi Y Chen M Tian L Xie J Long-term exercise prevents hepatic steatosis: a novel role of FABP1 in regulation of autophagy-lysosomal machinery. FASEB J (2019) 33(11):11870–83. 10.1096/fj.201900812R PMC6902714 31366243

[118] Bagherniya M Butler AE Barreto GE Sahebkar A The effect of fasting or calorie restriction on autophagy induction: A review of the literature. Ageing Res Rev (2018) 47:183–97. 10.1016/j.arr.2018.08.004 30172870

[119] Martinez-Lopez N Tarabra E Toledo M Garcia-Macia M Sahu S Coletto L System-wide Benefits of Intermeal Fasting by Autophagy. Cell Metab (2017) 26(6):856–71. 10.1016/j.cmet.2017.09.020 PMC5718973 29107505

[120] Madeo F Carmona-Gutierrez D Hofer SJ Kroemer G Caloric Restriction Mimetics against Age-Associated Disease: Targets, Mechanisms, and Therapeutic Potential. Cell Metab (2019) 29(3):592–610. 10.1016/j.cmet.2019.01.018 30840912

[121] Lin C-W Zhang H Li M Xiong X Chen X Chen X Pharmacological promotion of autophagy alleviates steatosis and injury in alcoholic and non-alcoholic fatty liver conditions in mice. J Hepatol (2013) 58(5):993–9. 10.1016/j.jhep.2013.01.011 PMC3634371 23339953

[122] Sinha RA You S-H Zhou J Siddique MM Bay B-H Zhu X Thyroid hormone stimulates hepatic lipid catabolism via activation of autophagy. J Clin Investigat (2012) 122(7):2428–38. 10.1172/JCI60580 PMC3386813 22684107

[123] Yan H Gao Y Zhang Y Inhibition of JNK suppresses autophagy and attenuates insulin resistance in a rat model of nonalcoholic fatty liver disease. Mol Med Rep (2017) 15(1):180–6. 10.3892/mmr.2016.5966 PMC5355648 27909723

[124] Song H Chu Q Yan F Yang Y Han W Zheng X Red pitaya betacyanins protects from diet-induced obesity, liver steatosis and insulin resistance in association with modulation of gut microbiota in mice. J Gastroenterol Hepatol (2016) 31(8):1462–9. 10.1111/jgh.13278 26699443

[125] Chu Q Zhang S Chen M Han W Jia R Chen W Cherry Anthocyanins Regulate NAFLD by Promoting Autophagy Pathway. Oxid Med Cell Longev (2019) 2019:4825949. 10.1155/2019/4825949 30931080 PMC6410467

[126] Allaire M Rautou P-E Codogno P Lotersztajn S Autophagy in liver diseases: Time for translation? J Hepatol (2019) 70(5):985–98. 10.1016/j.jhep.2019.01.026 30711404

[127] Kwanten WJ Martinet W Michielsen PP Francque SM Role of autophagy in the pathophysiology of nonalcoholic fatty liver disease: a controversial issue. World J Gastroenterol (2014) 20(23):7325–38. 10.3748/wjg.v20.i23.7325 PMC4064078 24966603

[128] Yu S Wang Z Ding L Yang L The regulation of TFEB in lipid homeostasis of non-alcoholic fatty liver disease: Molecular mechanism and promising therapeutic targets. Life Sci (2020) 246:117418. 10.1016/j.lfs.2020.117418 32057899

[129] Nakade Y Murotani K Inoue T Kobayashi Y Yamamoto T Ishii N Ezetimibe for the treatment of non-alcoholic fatty liver disease: A meta-analysis. Hepatol Res (2017) 47(13):1417–28. 10.1111/hepr.12887 28257594

